# Nicotine Exposure in the U.S. Population: Total Urinary Nicotine Biomarkers in NHANES 2015–2016

**DOI:** 10.3390/ijerph19063660

**Published:** 2022-03-19

**Authors:** Shrila Mazumder, Winnie Shia, Patrick B. Bendik, Honest Achilihu, Connie S. Sosnoff, Joseph R. Alexander, Zuzheng Luo, Wanzhe Zhu, Brittany N. Pine, June Feng, Benjamin C. Blount, Lanqing Wang

**Affiliations:** 1 Tobacco and Volatiles Branch, Division of Laboratory Sciences, National Center for Environmental Health, Centers for Disease Control and Prevention, Atlanta, GA 30341, USA; winnieshia@gmail.com (W.S.); obz3@cdc.gov (P.B.B.); wuv5@cdc.gov (H.A.); css3@cdc.gov (C.S.S.); or chemrick002@gmail.com (J.R.A.); huv0@cdc.gov (Z.L.); poq5@cdc.gov (W.Z.); pine.brittany@gmail.com (B.N.P.); czf2@cdc.gov (J.F.); bkb3@cdc.gov (B.C.B.); lfw3@cdc.gov (L.W.); 2 Oak Ridge Institute for Science and Education (ORISE), Oak Ridge, TN 37830, USA

**Keywords:** nicotine biomarkers, nicotine metabolites, total nicotine equivalents (TNE), urine, tobacco user, non-user, NHANES, exposure

## Abstract

We characterize nicotine exposure in the U.S. population by measuring urinary nicotine and its major (cotinine, trans-3′-hydroxycotinine) and minor (nicotine 1′-oxide, cotinine N-oxide, and 1-(3-pyridyl)-1-butanol-4-carboxylic acid, nornicotine) metabolites in participants from the 2015–2016 National Health and Nutrition Examination Survey. This is one of the first U.S. population-based urinary nicotine biomarker reports using the derived total nicotine equivalents (i.e., TNEs) to characterize exposure. Serum cotinine data is used to stratify tobacco non-users with no detectable serum cotinine (−sCOT), non-users with detectable serum cotinine (+sCOT), and individuals who use tobacco (users). The molar concentration sum of cotinine and trans-3′-hydroxycotinine was calculated to derive the TNE2 for non-users. Additionally, for users, the molar concentration sum of nicotine and TNE2 was calculated to derive the TNE3, and the molar concentration sum of the minor metabolites and TNE3 was calculated to derive the TNE7. Sample-weighted summary statistics are reported. We also generated multiple linear regression models to analyze the association between biomarker concentrations and tobacco use status, after adjusting for select demographic factors. We found TNE7 is positively correlated with TNE3 and TNE2 (r = 0.99 and 0.98, respectively), and TNE3 is positively correlated with TNE2 (r = 0.98). The mean TNE2 concentration was elevated for the +sCOT compared with the −sCOT group (0.0143 [0.0120, 0.0172] µmol/g creatinine and 0.00188 [0.00172, 0.00205] µmol/g creatinine, respectively), and highest among users (33.5 [29.6, 37.9] µmol/g creatinine). Non-daily tobacco use was associated with 50% lower TNE7 concentrations (*p* < 0.0001) compared with daily use. In this report, we show tobacco use frequency and passive exposure to nicotine are important sources of nicotine exposure. Furthermore, this report provides more information on non-users than a serum biomarker report, which underscores the value of urinary nicotine biomarkers in extending the range of trace-level exposures that can be characterized.

## 1. Introduction

Tobacco use is the leading preventable cause of disease, disability, and death in the United States. Each year, more than 400,000 deaths are attributed to cigarette smoking and exposure to secondhand smoke (SHS) [1]. The overall cigarette smoking rate has declined from 20.9% in 2005 to 15.5% in 2016; however, in 2016, nearly 38 million American adults continued to smoke cigarettes every day or some days [2]. Moreover, there are large disparities in smoking and exposure to SHS across different demographic groups, with people living below the poverty level and lower education attainment having the highest rate of cigarette smoking and SHS exposure among the general population [1]. In recent years, the U.S. Surgeon General also concluded secondhand aerosol (SHA) exposure from e-cigarettes to be not harmless [3], potentially exposing bystanders to nicotine and harmful constituents such as heavy metals, ultrafine particulates, volatile organic compounds, and other toxicants [4,5].

Nicotine (NIC) is an abundant alkaloid found in the leaves of tobacco plants. Being a highly addictive chemical, it is the main cause for continued tobacco use and contributes to the difficulty of quitting. Because NIC has a short elimination half-life in the body (0.5–3 h) [6], a more comprehensive way to estimate exposure is to measure nicotine metabolites that have longer elimination half-lives. The two predominant NIC metabolites in serum and urine are cotinine (COT) and trans-3′-hydroxycotinine (HCT), both of which have longer elimination half-lives compared to NIC—15–20 h for COT [7] and 6–9 h for HCT, after conversion from COT [8]. Minor nicotine metabolites, such as nicotine 1′-oxide (NOX), cotinine N-oxide (COX), and 1-(3-pyridyl)-1-butanol-4-carboxylic acid (HPB), are also present in substantial quantities in the urine collected from individuals who use tobacco [9]. Nornicotine (NNC), another minor tobacco biomarker, is both a constituent of tobacco leaves and a nicotine metabolite, and most urinary NNC is derived from metabolism of NIC, with less than 40% coming directly from tobacco [10]. Total nicotine equivalents (TNE) are the summed molar concentrations of the unconjugated and conjugated forms (“total”) of NIC and the metabolites. Partial conjugation of NIC and most of its metabolites to the *N*-glucuronide form—and HCT to the *O*-glucuronide form—is completed by multiple uridine 5′-diphospho-glucuronosyltransferase enzymes [9], leading to significant inter-individual differences in the glucuronidation rates of NIC and its metabolites [9]. Summing NIC and the six metabolites reported in this manuscript accounts for ~85% to 90% of the nicotine dose and is not significantly affected by individual differences in metabolism [11]. As such, TNEs provide a more complete assessment of nicotine exposures than NIC or COT alone.

The present study examined nicotine exposure in participants of the 2015–2016 National Health and Nutrition Examination Survey (NHANES) to obtain population-based biomonitoring data of the U.S. civilian, non-institutionalized population. Serum COT results from the same cycle of NHANES was used to stratify tobacco non-users and individuals who use tobacco. The results presented in this report provide a summary of the urinary concentrations of COT, HCT, and the TNEs, in both the tobacco user and non-user populations. The molar concentration sum of cotinine and trans-3′-hydroxycotinine was calculated to derive the TNE2 for non-users. For users, the molar concentration sum of nicotine and TNE2 was calculated to derive the TNE3, and the molar concentration sum of the minor metabolites and TNE3 was calculated to derive the TNE7. We also analyzed the association between nicotine biomarker concentrations and active tobacco use or passive nicotine exposure status after adjusting for sex, age, race/Hispanic origin, and education attainment. In addition, we calculated the least-square mean ratios using regression analysis to investigate any differences in exposure in relation to sex, age, race/Hispanic origin, and education attainment, after stratifying for tobacco use status. These data characterize nicotine exposure in the U.S. population for 2015–2016 and provide a baseline for future comparisons.

## 2. Materials and Methods

### 2.1. Study Design

The NHANES survey has been conducted by the National Center for Health Statistics (NCHS), a division of the U.S. Centers for Disease Control and Prevention (CDC), periodically since 1971 and continuously in two-year cycles since 1999. NHANES is a program of cross-sectional studies designed to assess the health and nutritional status of non-institutionalized U.S. civilians based on data collected from questionnaires, physical examinations, and biological samples [12]. The NCHS Research Ethics Review Board reviewed and approved the study (NCHS ERB Protocol #2011-17). Participants aged ≥18 provided informed written consent before taking part in the study. Participants < 18 years obtained parental permission, and documented assent for children and adolescents aged 7–17 was required, before taking part in the study. We measured nicotine and its metabolites in one-third of the spot urine samples from participants aged ≥6 years (NHANES datasets UCOT_I, COT_I; *n* = 3279). As laboratory examination components are carried out on a subsample of NHANES participants, NHANES 2015–2016 participants for some, but not all, ages were selected to provide urine samples for testing for nicotine metabolites. Each subsample is selected to be a nationally representative sample of the target population and has its own designated sample weight that accounts for the additional probability of selection into the subsample component. The results reported here are from a subset of these participants (*n* = 2281) remaining after applying eligibility criteria and discarding records with incomplete data.

### 2.2. Chemical Analysis

Nicotine biomarkers were measured by one of two separate isotope dilution liquid chromatography tandem mass spectrometry (LC-MS/MS) methods. We measured NIC and its six metabolites in urine samples with a total COT concentration of ≥20 µg/L (“high samples”) [13], and for urine samples with a total COT < 20 µg/L (“low samples”); only COT and HCT were measured [14]. The limit-of-detection (LOD) for NIC and its minor metabolites ranged from 1.38–10.5 µg/L, whereas the LOD for COT and HCT was determined to be 0.030 µg/L for both metabolites. Measurements below the LOD were substituted with the quotient of the LOD divided by the square root of two [15].

Briefly, urine aliquots were fortified with a labeled internal standard mixture and then incubated with beta-glucuronidase enzyme to hydrolyze the conjugated analytes. Samples were extracted and the nicotine biomarkers were measured by high-performance LC-MS/MS using electrospray ionization for high samples or ultra-high-performance LC-MS/MS using atmospheric-pressure chemical ionization for low samples. We monitored one quantitation transition, one confirmation transition, and one corresponding internal standard transition for each analyte quantified. Analyte concentrations were derived from the ratios of native-to-labeled compounds in the sample by comparing to a standard curve. Reported results met the accuracy and precision specifications of the quality control and quality assurance programs of CDC’s National Center for Environmental Health, Division of Laboratory Sciences [16].

### 2.3. Data Attrition and General Description of Dataset

**Scheme 1:**
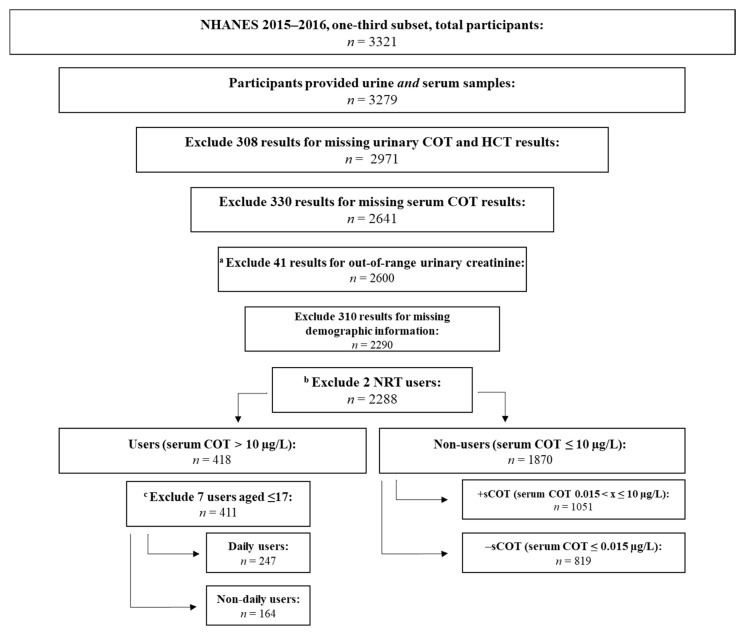
Data attrition and general description of non-user and user populations, in NHANES 2015–2016 (*n* = 2281). COT = cotinine; HCT = trans-3′-hydroxycotinine; NRT = nicotine replacement therapy; −sCOT = non-users with undetectable serum COT; +sCOT = non-users with detectable serum COT. ^a^: Samples with urinary creatinine concentrations outside of the 10–370 mg/dL range indicated excessively diluted or concentrated (in vivo) urine samples. ^b^: Users of NRT products were excluded from the analysis if participants indicated “yes” to the NHANES question SMQ863, within the SMQRTU_I questionnaire set. ^c^: Seven participants under the age of 17 were excluded due to the small sample size of this age group. For the user population, the steps taken to categorize daily and non-daily users have been provided in text.

Scheme 1 provides a summary of the data attrition process. Briefly, a total of 3321 participants were examined, of which 42 had provided no laboratory results. An additional 308 participants with missing urinary COT and HCT results and 330 with missing serum COT results were excluded from further data analysis. Participants with missing demographics information (*n* = 310) were also excluded, leaving a total of 2290 participant for additional attrition steps.

To distinguish non-users from those who use tobacco, we used a serum COT threshold of >10 µg/L, which has been identified as consistent with the active use of combusted cigarette products [17]. Among samples with serum COT ≤ 10 µg/L, those with serum COT less than or equal to the reported LOD (0.015 µg/L) were categorized as non-users with undetectable serum COT (“−sCOT”), and those with serum COT within 0.015 < x ≤ 10 µg/L were categorized as non-users with detectable serum COT (“+sCOT”) [18]. Responses from the NHANES questionnaire set, “Smoking—Recent Tobacco Use” (SMQRTU_I), were used to further categorize recent (within the past five days), daily, and non-daily tobacco users. Daily users are participants with serum COT > 10 µg/L who had reported using tobacco (at least one product type) daily within the past five days. Non-daily users are participants with serum COT > 10 µg/L who had reported using tobacco (any one product, or a combination of multiple products) for at least one day and up to four days, within the past five days. Within the SMQRTU_I dataset, the following NHANES questions for product usage frequency were used to categorize recent daily and non-daily users—SMQ710 (cigarettes), SMQ740 (pipes), SMQ770 (cigars), SMQ845 (hookah/water pipes), SMQ849 (e-cigarettes), SMQ800 (chewing tobacco), SMQ817 (snuff), SMQ857 (snus) and SMQ861 (dissolvables).

Table 1 shows the sample sizes and sample-weighted distributions for demographic groups stratified by tobacco use status for the 2281 participants included in this study. Self-reported information on sex, age, race/Hispanic origin, and education was collected by interview. Race/Hispanic origin was categorized as “non-Hispanic White”, “non-Hispanic Black”, “Hispanic” (participants identifying as “other Hispanic” or “Mexican American”), and “Other/Multiracial” (participants identifying as “non-Hispanic Asian”, “other race”, or “multiracial”). Age, in years, was divided into 18–29, 30–44, 45–59, and ≥60 for non-users and users; age categories of 6–11 and 12–17 were included for non-users only. Education attainment was defined based on the highest level of education completed, and categorized as “less than high school”, “high school graduate”, “some college (no degree)”, and “Bachelor’s degree or above”. The weighted urinary COT detection rates among each population sub-group were calculated as the percentage of measured analyte concentrations at or above the LOD. The COT detection rates are indicated in the superscript for each population sub-group.

### 2.4. Statistical Analysis

NHANES recruited participants through a multistage, probability sampling design involving selection of primary sampling units in counties, households in the counties, and sample patients in selected households [19]. Using this dataset, we calculated nationally representative summary statistics with appropriate variance estimates and investigated the associations of select demographic factors on nicotine exposure levels by applying survey sample weights (NHANES Subsample A Weight, WTSA2YR) and using Taylor series linearization for variance estimation. We used this estimation approach as it was implemented in the SURVEYFREQ, SURVEYMEANS, and SURVEYREG subroutines of the SAS^®^ statistical software application version 9.4 (SAS Institute, Cary, NC, USA). An evaluation of statistical reliability was performed to ensure all proportions followed NCHS Data Presentation Standards [20].

The Pearson correlation coefficients (r) and their *p*-values were computed between COT, HCT, NIC, TNE2, TNE3, and TNE7, where statistical significance was set to α ≤ 0.05. Pearson correlation coefficients were calculated from the log-transformed (base 10) biomarker and TNE concentrations without using sample-weights or adjusting the data for creatinine.

Creatinine concentration data were used to normalize the concentrations of nicotine exposure biomarkers to account for urine volume variability and the variability in concentrations of endogenous and exogenous chemicals [21]. Summary statistics, including sample-weighted geometric means (GM) of biomarkers and TNEs, along with their 95% confidence intervals (CI), are reported as a ratio of creatinine (µg/g creatinine, or µmol/g creatinine) in the main tables and volume-weighted concentrations (µg/L, or µmol/L) in the Supplementary Tables S1 and S2.

Sample-weighted multiple linear regression models stratified by tobacco use status were fitted to data from the NHANES 2015–2016 cycle, where the dependent factors were the creatinine-unadjusted concentrations of COT, HCT, TNE2, TNE3, and TNE7, and the independent factors included both continuous (creatinine, g/L) and categorical types (i.e., sex, age, race/Hispanic origin, education attainment, extent of passive nicotine exposure among non-users, and tobacco use frequency). Because the distribution of biomarker measurements was highly right-skewed—which would have adversely affected hypothesis testing—urinary creatinine, COT, HCT, and TNEs concentration data were log-transformed (base 10) to enable evaluation of the statistical significance of regression coefficients. We report the exponentiated coefficients from these models along with their 95% CIs and *p*-values, where statistical significance was set to α ≤ 0.05. The exponentiated coefficients represent the proportional change of biomarker concentration associated with an independent categorical or continuous predictor. To interpret the categorical factors in the model, the associated percentage difference in biomarker concentration was calculated as the exponentiated coefficient minus 1 and then multiplied by 100.

For the regression models, we accounted for urinary dilution by including urinary creatinine as an independent factor. Among the other independent factors included in the model, we used the following categories as reference groups: males for sex, “45–59” years for age group, “non-Hispanic White” for race/Hispanic origin, “Bachelor’s degree or above” for education attainment group, “+sCOT” for non-user sub-group and “daily user” for user sub-group. Regarding our regression analyses on the education attainment group, to ensure that none of the younger participants in the main analysis were misclassified due to being “too young” to have attained their highest degree at the time of the survey, we performed a sub-analysis of adults aged 25 or older in separate sample-weighted log-linear regression models after stratifying for tobacco use status (Supplementary Tables S5 and S6). We found no difference in significance of education attainment between our main analyses and the sub-analyses. We also performed pairwise comparisons of least-square means from the regressions, among different demographic groups, for both the non-user and user populations (Supplementary Tables S3 and S4). To correct for multiple comparisons, we adjusted the *p*-values from pairwise comparisons by the Bonferroni method.

## 3. Results

### 3.1. Correlation of Nicotine Biomarkers and TNEs

**Figure 1:**
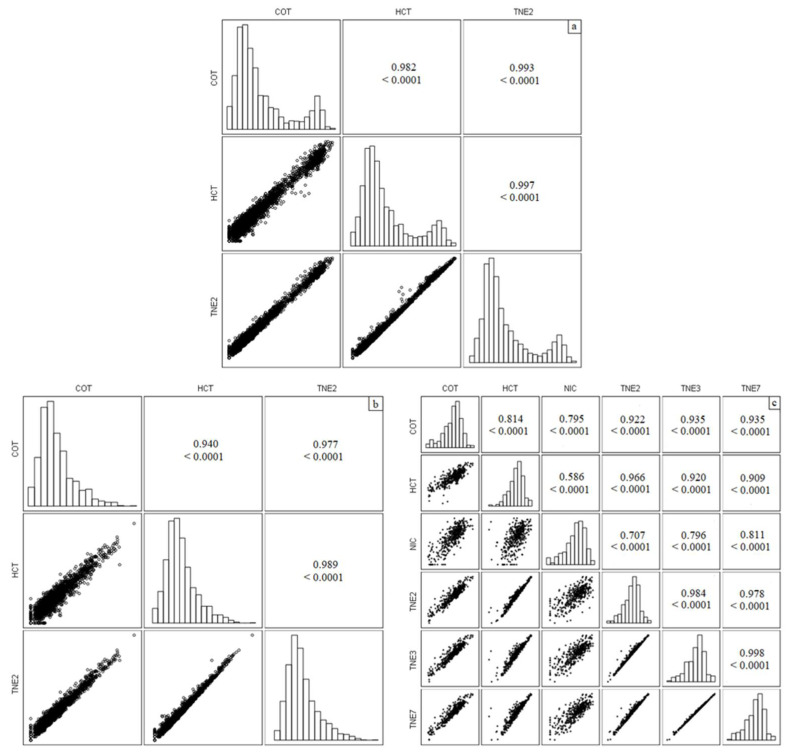
Logarithmic distributions and correlations for urinary cotinine, trans-3′-hydroxycotinine, nicotine, and TNEs, from NHANES 2015–2016. Within each panel, the Pearson correlation coefficient is designated as the top number, and the *p*-value is designated as the bottom number. Pearson correlation coefficients are obtained from un-weighted, log-transformed (base 10) biomarker and TNE concentrations without adjusting for the urinary creatinine concentration. Each panel contains information for (**a**) combined non-user and user populations, (**b**) non-user population only, and (**c**) user population only.

Correlation plots were generated to determine the strength of associations between nicotine and its major metabolites and the TNEs (Figure 1). Among users, TNE7 positively correlated with TNE3 and TNE2 (r = 0.99 and 0.98, respectively), and TNE3 positively correlated with TNE2 (r = 0.98). COT and HCT concentrations were strongly correlated in both user and non-user population sub-groups, with a higher degree of correlation recorded among the non-users (r = 0.94 vs. r = 0.81). TNE7 and TNE3 correlated very well with COT and HCT (r ≥ 0.91), though a slightly stronger correlation was found with COT (r = 0.94 and 0.93, respectively). TNE2 was strongly correlated to COT and HCT (r ≥ 0.92) within either population sub-groups, where we found a slightly higher degree of correlation between HCT and TNE2 (r = 0.97 and 0.99 among individuals who use tobacco and non-users, respectively).

### 3.2. Estimates of Nicotine Exposure by TNE2 and TNE7 in the U.S. Population

We found similar TNE2 concentrations for each demographic group of −sCOT (Table 2), with a relatively large range in biomarker concentrations noted when this population sub-group was categorized by age. TNE2 ranged from 0.00151 [0.00118, 0.00192] µmol/g creatinine in the 12–17 age group, to 0.00218 [0.00182, 0.00261] µmol/g creatinine in the 6–11 age group. The “non-Hispanic Black” group had the lowest exposure (0.00154 [0.00125, 0.00190] µmol/g creatinine) when compared with other race/Hispanic origin groups (0.00187 [0.00158, 0.00220] µmol/g creatinine to 0.00191 [0.00171, 0.00214] µmol/g creatinine). Categorizing based on sex showed females to have 23% higher exposure than males (0.00205 [0.00187, 0.00224] µmol/g creatinine and 0.00166 [0.00147, 0.00188] µmol/g creatinine, respectively). We recorded similar TNE2 (0.00181 [0.00161, 0.00203] µmol/g creatinine to 0.00196 [0.00163, 0.00235] µmol/g creatinine) when categorizing −sCOT by education attainment.

The TNE2 concentration for all +sCOT was higher when compared with all −sCOT (0.0143 [0.0120, 0.0172] µmol/g creatinine and 0.00188 [0.00172, 0.00205] µmol/g creatinine, respectively) (Table 2). Among +sCOT, categorized by age, the peak level of exposure was within the 6–11 age group (0.0252 [0.0194, 0.0329] µmol/g creatinine), whereas the 30–44 age group had the lowest level of exposure (0.00958 [0.00675, 0.0136] µmol/g creatinine). Overall, a slight decrease in exposure among those aged 30 and above was found within the +sCOT sub-group. We noted lower TNE2 as education level increased from “high school graduate” (0.0272 [0.0180, 0.0413] µmol/g creatinine) to “Bachelor’s degree or above” (0.00780 [0.00514, 0.0118] µmol/g creatinine). Among males and females, similar TNE2 (0.0145 [0.0110, 0.0192] µmol/g creatinine and 0.0141 [0.0117, 0.0171] µmol/g creatinine, respectively) were recorded. The “Other/Multiracial” group had the lowest exposure (0.0100 [0.00722, 0.0139] µmol/g creatinine) when compared with other race/Hispanic origin groups (0.0142 [0.0115, 0.0176] µmol/g creatinine to 0.0154 [0.0117, 0.0203] µmol/g creatinine).

Total concentrations of the full panel of analytes were measured for users to calculate the TNE7, in addition to the TNE2 and TNE3 (Table 3). TNE7 for daily users was higher than for non-daily users (65.7 [55.6, 77.7] µmol/g creatinine and 25.6 [19.6, 33.4] µmol/g creatinine, respectively). The youngest individuals who used tobacco had the lowest exposure (20.2 [16.5, 24.6] µmol/g creatinine) when compared with the older age groups. Nicotine exposure peaked in the 45–59 age group (76.7 [63.3, 92.8] µmol/g creatinine) and then fell to a level 30% lower in the oldest age group (55.9 [46.4, 67.4] µmol/g creatinine). TNE7 decreased as education attainment increased from “less than high school” (55.5 [46.7, 66.0] µmol/g creatinine) to “Bachelor’s degree or above” (38.4 [30.5, 48.5] µmol/g creatinine). Nicotine exposure for females was 15% higher than for males (50.4 [42.7, 59.6] µmol/g creatinine and 43.8 [38.9, 49.3] µmol/g creatinine, respectively). Non-Hispanic Whites had the highest exposure levels (59.7 [52.1, 68.4] µmol/g creatinine) and Hispanics had the lowest (24.2 [16.9, 34.7] µmol/g creatinine), with Non-Hispanic Blacks having similar exposure levels (25.7 [20.8, 31.7] µmol/g creatinine) as the latter group.

### 3.3. Factors Influencing Nicotine Exposure in the U.S. Population

Results in this sub-section are from multiple linear regressions of logarithmic COT, HCT, or TNEs on non-user and user sub-groups, controlled for urinary creatinine and the demographic factors sex, age, race/Hispanic origin, and education attainment. The results presented in Table 4 and Table 5 were obtained after including all participants aged ≥6 years for non-users and ≥18 years for individuals who use tobacco, respectively. In all regression models, urinary creatinine had a small but statistically significant (*p* < 0.0001) association with the individual biomarker and TNE concentrations.

Among non-users, −sCOT had 86% lower TNE2 compared with non-users having higher nicotine exposure (*p* < 0.0001) (Table 4). Demographic factors, including race/Hispanic origin, and education attainment, also had statistically significant associations with nicotine exposure among non-users. Compared to non-Hispanic Whites, Hispanics had 19% lower TNE2 (*p* = 0.0114), and the “Other/Multiracial” group had 29% lower HCT (*p* = 0.029). Non-users without a high school degree and those with a high school degree were found to have higher TNE2 (68%, *p* = 0.004 and 120%, *p* < 0.0001, respectively) when compared with those having a Bachelor’s (or higher) degree. Individuals with some college education had 33% higher HCT compared with those having a Bachelor’s (or higher) degree (*p* = 0.0351). Neither sex nor age had any association with the TNE2 concentrations; however, we found the 18–29 age group had higher COT than the 45–59 age group (55%, *p* = 0.0462).

Among individuals who use tobacco, non-daily users had 50% lower TNE7 compared with daily users (*p* < 0.0001) (Table 5). Demographic factors, including age and race/Hispanic origin, were also statistically significantly associated with nicotine exposure among users. Compared to the 45–59 age group, those aged 18–29, 30–44, and ≥60 had lower TNE7 (67%, *p* < 0.0001, 35%, *p* = 0.0065 and 28%, *p* = 0.0309, respectively). The “Hispanic”, “non-Hispanic Black”, and “other/multiracial” groups had lower TNE7 (47%, *p* = 0.0009; 48%, *p* < 0.0001; and 34%, *p* = 0.0128, respectively), when compared to the “non-Hispanic White” group. Neither sex nor education attainment had any association with nicotine exposure among individuals who use tobacco.

## 4. Discussion

We measured COT, HCT, and TNEs in urine samples from a one-third subset of NHANES 2015–2016 cycle, aged ≥6. Our regression models show that tobacco use frequency and passive exposure to nicotine are important sources of nicotine exposure in the U.S. population. After controlling for the extent of passive nicotine exposure, creatinine, and other demographic factors, we find that non-Hispanic Whites tended to have higher urinary TNE2 than other race/ethnicities, with this difference reaching statistical significance for Hispanics. Furthermore, education attainment was inversely associated with urinary TNE2 levels: attaining a Bachelor’s (or higher) degree was associated with less nicotine exposure than that found in people with lower educational attainment. Among individuals who use tobacco, demographic factors were also evaluated for association with nicotine exposure in the sample-weighted multiple linear regression models. After controlling for tobacco use frequency, creatinine, and other demographic factors, we find that non-Hispanic Whites had significantly higher urinary TNE7 compared with other race/ethnicities. Additionally, urinary TNE7 was higher in the 45–59 age group compared with any of the other age groups.

A key strength of our study was the use of measured concentrations of multiple, well-established nicotine metabolites, rather than the use of a single metabolite or product use questions alone. Another major strength of this study is the use of biochemical verification of tobacco use status. We confirmed passive nicotine exposure status using participants’ serum COT concentrations, where we were able to compare the urinary COT, HCT, and TNE2 concentrations between −sCOT and +sCOT and provide reference ranges for the biomarkers among the two population sub-groups. We noted good agreement between the COT and TNE2 concentrations in our population estimates and regression analysis, which further supports the utility of either one of these biomarkers for monitoring and assessing exposure levels within the two population sub-groups. Recent active use of tobacco products was confirmed by the serum COT > 10 µg/L cutoff and responses to the product use survey questionnaire. By accounting for questionnaire responses regarding past-five-day use of single or multiple tobacco products, along with participant serum COT concentrations, we were also able to remove some degree of uncertainty in identifying non-daily users. The past-five-day responses were deemed more appropriate than responses from the past 30 days usage questionnaire for identifying non-daily users because the metabolite half-lives are relatively short. Moreover, as this study used nationally representative data, our results provide reliable measures of nicotine exposure among the U.S. population.

Within non-users, we see quantitative differences in TNE2 GMs among the two population sub-groups and some of the demographic groups. As anticipated, the TNE2 GM for the +sCOT sub-group was elevated when compared to −sCOT. Within +sCOT, higher TNE2 among those aged 6–11 years than those aged ≥12 years could result from a larger proportion of younger children being exposed to higher levels of SHS and/or SHA than youths and adults. For anyone living with one or more family members who smoke tobacco, one can expect children, who are generally spending more time within their homes, to have a greater propensity for exposure to SHS and/or SHA [22,23]. Within the same population sub-group, individuals with lower education attainment tended to have higher TNE2, whereas those with higher education attainment had lower biomarker concentrations. A possible explanation for such exposure pattern could be that people with lower education attainment may be less aware of the health hazards of smoking, SHS, and SHA, and thus have a greater propensity for exposure. It may be of interest to further stratify this population sub-group by perceived SHS or SHA exposure to note any substantial differences in biomarker concentrations and track such information across multiple NHANES cycles.

The GMs of urinary nicotine metabolites and TNEs varied by age, sex, race/Hispanic origin and education attainment among individuals who use tobacco, where the exposure patterns were generally consistent with those from previous studies. Advanced age was associated with higher nicotine exposure, and we generally noted similar patterns in exposure across the different age groups when using either TNEs or COT. An overall pattern of increasing biomarker concentration by age was also reported in other studies [24,25]. Possible explanations for such a pattern may include differences in tobacco use frequency, differences in the intensity in smoking behavior, and/or lower representation of light users among the older age groups. Creatinine adjustment has a well-known impact on sex differences, as males, on average, have ~30% higher urinary creatinine concentrations than females [26]. As such, creatinine adjustment has a predictable influence on the reported GMs, as shown by the 3–15% higher nicotine metabolite and TNE concentrations among female users. When categorizing by race/Hispanic origin, our study extends the literature—in which, serum COT was generally used for biomonitoring [27,28], followed by TNEs [24]—by presenting users identifying as Hispanic or Mexican American to have lower concentrations of urinary biomarkers than non-Hispanic Whites and non-Hispanic Blacks. We also find that non-Hispanic Whites had higher urinary nicotine biomarkers compared with other race/Hispanic origin groups, perhaps because the tobacco use group included people who use smokeless tobacco. Smokeless tobacco use is associated with higher nicotine exposure compared with other tobacco products [24,29,30,31], and smokeless tobacco users are disproportionately non-Hispanic White [29,30]. Higher education attainment among individuals who use tobacco was not associated with lower nicotine exposure in the weighted multiple linear regression models; however, we note an overall pattern in decreasing exposure levels among the higher education attainment groups, which generally followed the pattern noted in previous studies using serum COT [27] and urinary TNEs [24].

Several recent studies have characterized non-Hispanic Black cigarette smokers as having higher exposure levels than non-Hispanic Whites [27,28,32,33] when comparing their serum COT or urinary COT (unconjugated) concentrations. Other studies that accounted for total urinary NIC and its two major metabolites generally found higher exposure levels among non-Hispanic White tobacco users [24] and exclusive cigarette users [25] compared with non-Hispanic Black users, which is consistent with the results presented in this report. The above observations may potentially reflect a difference in the type of biomarker used to characterize the population exposure levels (i.e., unconjugated vs. total measurements), rather than any inherent differences between the study type. In addition, non-Hispanic Blacks are reported to have lower COT (and NIC) glucuronidation rates compared to non-Hispanic Whites [34,35], which may explain the higher serum COT concentrations—and further explain the lower urinary COT concentrations—reported for non-Hispanic Blacks compared to non-Hispanic Whites. Because substantial racial/ethnic differences are observed in nicotine metabolism [36,37,38]—which may be influenced by both genetic and environmental factors [39,40]—the use of a nicotine metabolite that is susceptible to metabolic-related differences may not be comprehensive in characterizing exposure levels within large, population-wide studies.

We analyzed nicotine metabolites in both urine and serum collected from the same NHANES study participants. COT in these two matrices was highly correlated, which is consistent with urine/blood measure correlations in other studies (r = 0.69 to 0.91, *p* < 0.05) [41,42,43]. Importantly, COT concentrations are typically 4- to 5-fold higher in urine compared with blood plasma or serum [42]; the difference between urine and blood matrices is mostly attributable to renal clearance processes [9]. These findings underscore the value of urinary nicotine biomarkers in extending the range of trace-level nicotine exposures that can be characterized. In our current dataset, for example, COT was detected in 94% of non-user urine samples collected from study participants with serum COT that was ≤0.015 µg/L. The high COT detection rate obtained for the urine samples analyzed would suggest persistent exposure to nicotine in the U.S. population; however, such an observation would not be as apparent when comparing the serum detection rate from the same population sub-group.

Among urinary biomarkers of nicotine exposure, urinary TNEs are better suited for characterizing nicotine exposure than any single metabolite, such as COT. Our analysis showed strong TNE2-COT and TNE2-HCT correlations (r ≥ 0.92), among both the non-user and user populations, and very strong correlations between TNE2, TNE3, and TNE7 (r ≥ 0.98) when surveying the user population. Among non-users, TNE2 could estimate nicotine exposure reasonably well, as the molar sum of COT and HCT typically accounts for ~70% of the total nicotine dose [9]. TNE2 may also be sufficient for estimating active usage, as it is strongly correlated to both TNE3 and TNE7. TNE3 or TNE7 would be good biomarkers to estimate exposure among individuals who use tobacco; however, TNE7 would provide the best estimate of recent nicotine exposure as it accounts for ~85% to 90% of the total nicotine dose [11]. Overall, the use of TNEs may provide a more suitable assessment of nicotine exposure because these measurements are not significantly affected by individual differences in metabolism.

Some of the constraints in the current analysis included the limited sample size obtained for individuals who use tobacco after applying all necessary criteria for data analyses, and limited sample size obtained for users of tobacco products other than cigarettes. In addition, by using the serum COT cutoff to categorize the non-user and user populations, we introduced the potential for a portion of the self-reported non-daily users to be classified as non-users. Furthermore, misclassification resulting from misreporting of information in self-reported questionnaire responses is likely. Lastly, we measured nicotine exposure biomarkers with relatively short half-lives (15–20 h for the major metabolites) in a spot urine sample, and thus introduce a degree of imprecision for non-daily users due to potential variations in the time elapsed between last tobacco use and urine collection.

## 5. Conclusions

We characterized nicotine exposure among individuals who use tobacco and non-users with passive exposure to nicotine in a representative sample of the U.S. population, based on data collected from the NHANES 2015–2016 study cycle. This paper provides pertinent biomonitoring data to assess public health risk and identify population sub-groups that are at a higher risk of being exposed to tobacco. Our current analysis documents important differences in nicotine exposure and shows that, along with certain demographic factors such as age, race/Hispanic origin, and education attainment, tobacco use frequency and passive exposure to nicotine are major contributors to increased nicotine exposure. These data provide a crucial baseline against which future analyses of urinary nicotine biomarkers can be compared to document variations caused by changes in tobacco products, use behaviors, and/or policies/regulations.

## Figures and Tables

**Table 1:** Sample size, urinary cotinine detection rates, sample-weighted demographic distributions (%-population) and standard error (SE), in NHANES 2015–2016 (*n* = 2281).

	¹ All Non-Users			² −sCOT			³ +sCOT			⁴ All Users		
	Sample Size, *n*	%-Population	SE	Sample Size, *n*	%-Population	SE	Sample Size, *n*	%-Population	SE	Sample Size, *n*	%-Population	SE
All	1870	79.8	1.55	819	37.9	2.25	1051	41.5	1.52	411	20.8	1.55
Age (years)												
* 6−11	295	8.61	0.676	109	6.96	0.679	186	10.1	0.986			
* 12−17	271	10.9	0.734	98	7.89	0.988	173	13.6	0.878			
18−29	239	15.3	1.24	87	11.4	1.97	152	18.9	2.17	85	21	3.34
30−44	314	18.6	1.53	152	19	2.08	162	18.2	1.77	128	33.1	4.07
45−59	307	22.2	1.8	148	26.5	3.06	159	18.3	2.33	104	30.8	2.97
≥60	444	24.4	1.89	225	28.2	4.04	219	20.9	1.83	94	15.1	2.4
Race/Hispanic origin												
Non-Hispanic White	549	60.8	4.78	235	64.2	4.97	314	57.7	5.22	164	67.8	3.52
Non-Hispanic Black	315	9.41	1.88	87	6.23	1.34	228	12.3	2.56	124	16	2.87
Hispanic	679	19.1	3.71	362	21	4.71	317	17.4	3.06	85	10.8	2.25
Other/Multiracial	327	10.6	1.64	135	8.57	1.81	192	12.5	1.78	38	5.43	1.06
Sex												
Male	868	46.2	1.69	344	41.2	2.06	524	50.7	2.19	263	62.5	2.44
Female	1002	53.8	1.69	475	58.8	2.06	527	49.3	2.19	148	37.5	2.44
Education attainment												
Less than high school	856	29.7	1.94	347	24.9	2.57	509	34.2	1.97	115	21.7	3.17
High school graduate	274	14.4	1.24	98	11	1.65	176	17.4	1.61	123	29.5	1.74
Some college (no degree)	362	26	1.39	150	25	2.05	212	26.8	2.59	116	32.3	4.11
Bachelor’s degree or above	378	29.9	2.92	224	39.1	4.04	154	21.6	2.72	57	16.5	2.29
User												
Daily										247	62.4	3.16
Non-daily										164	37.6	3.16
Non-user												
−sCOT	819	47.7	2.25									
+sCOT	1051	52.3	2.25									

−sCOT = non-users with undetectable serum COT; +sCOT = non-users with detectable serum COT. Detection rates were calculated using weighted, urinary cotinine concentrations— ^1^: 96%; ^2^: 94%; ^3^: 99%; ^4^: 100%. *: Age categories of 6–11 and 12–17 were included for non-users only.

**Table 2:** Sample-weighted creatinine adjusted geometric mean for cotinine, trans-3′-hydroxycotinine (µg/g creatinine) and TNE2 (µmol/g creatinine), with 95% confidence interval, among non-users, from NHANES 2015–2016.

	−sCOT			+sCOT			All Non-Users		
	COT	HCT	TNE2	COT	HCT	TNE2	COT	HCT	TNE2
All	0.125 [0.115, 0.135]	0.211 [0.190, 0.233]	0.00188 [0.00172, 0.00205]	0.922 [0.770, 1.104]	1.64 [1.37, 1.97]	0.0143 [0.0120, 0.0172]	0.355 [0.297, 0.424]	0.616 [0.518, 0.733]	0.00543 [0.00455, 0.00647]
6−11	0.132 [0.106, 0.165]	0.259 [0.217, 0.309]	0.00218 [0.00182, 0.00261]	1.60 [1.18, 2.16]	2.92 [2.27, 3.76]	0.0252 [0.0194, 0.0329]	0.611 [0.480, 0.777]	1.15 [0.897, 1.47]	0.00982 [0.00775, 0.0124]
12−17	0.102 [0.0861, 0.121]	0.165 [0.120, 0.228]	0.00151 [0.00118, 0.00192]	0.971 [0.517, 1.83]	1.66 [0.871, 3.18]	0.0147 [0.00773, 0.0279]	0.446 [0.281, 0.706]	0.750 [0.469, 1.20]	0.00668 [0.00421, 0.0106]
18–29	0.139 [0.116, 0.167]	0.204 [0.172, 0.242]	0.00194 [0.00165, 0.00228]	1.46 [0.804, 2.65]	2.40 [1.24, 4.62]	0.0219 [0.0117, 0.0410]	0.633 [0.405, 0.990]	0.998 [0.609, 1.63]	0.00924 [0.00575, 0.0149]
30–44	0.129 [0.112, 0.148]	0.191 [0.164, 0.222]	0.00180 [0.00158, 0.00207]	0.632 [0.448, 0.893]	1.08 [0.760, 1.53]	0.00958 [0.00675, 0.0136]	0.291 [0.245, 0.344]	0.463 [0.380, 0.564]	0.00424 [0.00352, 0.00511]
45–59	0.126 [0.107, 0.148]	0.210 [0.169, 0.262]	0.00186 [0.00155, 0.00223]	0.753 [0.474, 1.20]	1.35 [0.865, 2.12]	0.0117 [0.00746, 0.0184]	0.271 [0.192, 0.383]	0.469 [0.330, 0.666]	0.00410 [0.00290, 0.00579]
≥60	0.121 [0.108, 0.136]	0.232 [0.209, 0.256]	0.00196 [0.00178, 0.00217]	0.750 [0.485, 1.16]	1.49 [0.994, 2.24]	0.0124 [0.00819, 0.0188]	0.274 [0.203, 0.370]	0.534 [0.403, 0.707]	0.00449 [0.00337, 0.00598]
Non-Hispanic White	0.127 [0.114, 0.141]	0.216 [0.188, 0.248]	0.00191 [0.00171, 0.00214]	0.994 [0.761, 1.30]	1.79 [1.34, 2.40]	0.0154 [0.0117, 0.0203]	0.352 [0.278, 0.446]	0.617 [0.489, 0.781]	0.00539 [0.00426, 0.00681]
Non-Hispanic Black	0.0853 [0.0675, 0.108]	0.188 [0.155, 0.228]	0.00154 [0.00125, 0.00190]	0.831 [0.631, 1.09]	1.77 [1.31, 2.40]	0.0149 [0.0112, 0.0197]	0.405 [0.290, 0.564]	0.871 [0.612, 1.24]	0.00728 [0.00518, 0.0102]
Hispanic	0.128 [0.115, 0.144]	0.208 [0.184, 0.237]	0.00188 [0.00168, 0.00210]	0.937 [0.757, 1.16]	1.60 [1.29, 1.99]	0.0142 [0.0115, 0.0176]	0.331 [0.278, 0.395]	0.551 [0.446, 0.681]	0.00492 [0.00405, 0.00599]
Other/Multiracial	0.136 [0.117, 0.159]	0.192 [0.158, 0.233]	0.00187 [0.00158, 0.00220]	0.707 [0.526, 0.951]	1.05 [0.726, 1.53]	0.0100 [0.00722, 0.0139]	0.375 [0.299, 0.471]	0.547 [0.413, 0.724]	0.00525 [0.00409, 0.00672]
Male	0.109 [0.0978, 0.122]	0.189 [0.165, 0.217]	0.00166 [0.00147, 0.00188]	0.950 [0.719, 1.25]	1.65 [1.25, 2.19]	0.0145 [0.0110, 0.0192]	0.378 [0.297, 0.481]	0.656 [0.514, 0.837]	0.00576 [0.00451, 0.00735]
Female	0.137 [0.124, 0.151]	0.227 [0.204, 0.252]	0.00205 [0.00187, 0.00224]	0.895 [0.743, 1.08]	1.63 [1.34, 1.99]	0.0141 [0.0117, 0.0171]	0.336 [0.285, 0.397]	0.584 [0.499, 0.683]	0.00516 [0.00441, 0.00605]
Less than high school	0.123 [0.111, 0.137]	0.220 [0.194, 0.249]	0.00192 [0.00173, 0.00212]	1.14 [0.817, 1.58]	1.99 [1.44, 2.73]	0.0175 [0.0126, 0.0241]	0.468 [0.351, 0.624]	0.825 [0.618, 1.10]	0.00722 [0.00542, 0.00962]
High school graduate	0.133 [0.108, 0.164]	0.211 [0.174, 0.256]	0.00196 [0.00163, 0.00235]	1.69 [1.12, 2.54]	3.18 [2.08, 4.87]	0.0272 [0.0180, 0.0413]	0.668 [0.472, 0.946]	1.18 [0.802, 1.74]	0.0104 [0.00719, 0.0151]
Some college (no degree)	0.122 [0.0983, 0.151]	0.220 [0.181, 0.267]	0.00191 [0.00158, 0.00231]	0.734 [0.518, 1.04]	1.41 [0.991, 2.01]	0.0120 [0.00847, 0.0169]	0.322 [0.238, 0.434]	0.600 [0.450, 0.799]	0.00514 [0.00384, 0.00689]
Bachelor’s degree or above	0.125 [0.113, 0.139]	0.199 [0.174, 0.228]	0.00181 [0.00161, 0.00203]	0.539 [0.355, 0.819]	0.861 [0.562, 1.32]	0.00780 [0.00514, 0.0118]	0.217 [0.176, 0.268]	0.345 [0.274, 0.435]	0.00314 [0.00253, 0.00389]

COT = cotinine; HCT = trans-3′-hydroxycotinine; TNE2 = (total cotinine/176.2151) + (total trans-3′-hydroxycotinine/192.2145); −sCOT = non-users with undetectable serum COT; +sCOT = non-users with detectable serum COT.

**Table 3:** Sample-weighted creatinine adjusted geometric mean for cotinine, trans-3′-hydroxycotinine (µg/g creatinine) and TNE2, TNE3, TNE7 (µmol/g creatinine), with 95% confidence interval, among people who use tobacco, from NHANES 2015–2016.

	COT	HCT	TNE2	TNE3	TNE7
All	2113 [1857, 2404]	3781 [3292, 4342]	33.5 [29.6, 37.9]	40.5 [35.6, 46.1]	46.2 [40.7, 52.3]
18−29	959 [757, 1215]	1577 [1230, 2022]	14.9 [12.0, 18.6]	17.6 [14.4, 21.7]	20.2 [16.5, 24.6]
30−44	2094 [1585, 2765]	3628 [2725, 4830]	32.2 [24.4, 42.5]	39.5 [29.9, 52.2]	44.6 [33.9, 58.7]
45−59	3394 [2756, 4179]	6466 [5359, 7801]	55.3 [46.5, 65.8]	67.5 [55.9, 81.5]	76.7 [63.3, 92.8]
≥60	2464 [1937, 3133]	4677 [4038, 5418]	40.2 [33.7, 48.0]	48.2 [40.1, 58.1]	55.9 [46.4, 67.4]
Non-Hispanic White	2780 [2433, 3176]	4936 [4265, 5713]	43.3 [38.0, 49.4]	52.7 [45.9, 60.4]	59.7 [52.1, 68.4]
Non-Hispanic Black	970 [803, 1170]	2176 [1636, 2895]	18.5 [14.9, 22.9]	22.1 [17.8, 27.5]	25.7 [20.8, 31.7]
Hispanic	1203 [815, 1776]	1940 [1146, 3283]	17.9 [11.8, 27.1]	21.2 [14.6, 30.9]	24.2 [16.9, 34.7]
Other/Multiracial	2089 [1068, 4086]	2591 [1347, 4986]	26.4 [13.9, 50.3]	32.9 [16.9, 63.8]	37.8 [19.7, 72.7]
Male	2039 [1780, 2337]	3733 [3265, 4269]	32.6 [28.7, 36.9]	38.5 [34.1, 43.6]	43.8 [38.9, 49.3]
Female	2242 [1926, 2610]	3861 [3067, 4860]	35.0 [29.6, 41.4]	44.0 [37.1, 52.3]	50.4 [42.7, 59.6]
Less than high school	2582 [2095, 3183]	4160 [3213, 5385]	38.8 [31.5, 47.8]	48.2 [40.3, 57.7]	55.5 [46.7, 66.0]
High school graduate	2323 [1776, 3039]	4135 [3254, 5253]	36.9 [29.1, 46.7]	44.3 [34.6, 56.8]	50.4 [39.2, 64.7]
Some college (no degree)	1862 [1411, 2458]	3703 [2920, 4695]	31.0 [24.1, 39.9]	36.5 [28.1, 47.5]	41.4 [31.9, 53.6]
Bachelor’s degree or above	1754 [1391, 2211]	2957 [2060, 4245]	26.9 [20.2, 35.9]	33.6 [26.5, 42.5]	38.4 [30.5, 48.5]
Daily	2999 [2486, 3618]	5227 [4347, 6284]	46.3 [39.0, 55.1]	57.5 [48.4, 68.3]	65.7 [55.6, 77.7]
Non-daily	1181 [897, 1554]	2206 [1622, 3000]	19.5 [14.7, 25.7]	22.6 [17.3, 29.7]	25.6 [19.6, 33.4]

COT = cotinine; HCT = trans-3′-hydroxycotinine; TNE2 = (total cotinine/176.2151) + (total trans-3′-hydroxycotinine/192.2145); TNE3 = (total nicotine/162.2316) + (total cotinine/176.2151) + (total trans-3′-hydroxycotinine/192.2145); TNE7 = (total nicotine/162.2316) + (total cotinine/176.2151) + (total trans-3′-hydroxycotinine/192.2145) + (total cotinine N-oxide/192.2145) + (total nicotine 1′-oxide/178.231) + (total 1-(3-pyridyl)-1-butanol-4-carboxylic acid/181.1885) + (total nornicotine/148.2050).

**Table 4:** Sample-weighted log-linear regression results for urinary cotinine (µg/L), trans-3′-hydroxycotinine (µg/L) and TNE2 (µmol/L) on extent of passive exposure to nicotine and demographic factors among non-users, from NHANES 2015–2016.

Factor	Level	COT	*p*-Value	HCT	*p*-Value	TNE2	*p*-Value
		Exponentiated Coefficient (95% CI)		Exponentiated Coefficient (95% CI)		Exponentiated Coefficient (95% CI)	
Intercept		0.292 [0.209, 0.406]	**<0.0001**	0.408 [0.305, 0.544]	**<0.0001**	0.004 [0.003, 0.005]	**<0.0001**
Creatinine, urine		1.007 [1.006, 1.008]	**<0.0001**	1.009 [1.007, 1.010]	**<0.0001**	1.008 [1.007, 1.009]	**<0.0001**
Non-user	−sCOT	0.143 [0.118, 0.173]	**<0.0001**	0.137 [0.112, 0.168]	**<0.0001**	0.140 [0.116, 0.169]	**<0.0001**
	+sCOT	Ref.		Ref.		Ref.	
Sex	Female	0.946 [0.799, 1.122]	0.4998	0.987 [0.824, 1.182]	0.876	0.979 [0.821, 1.166]	0.7957
	Male	Ref.		Ref.		Ref.	
Age	6−11	1.207 [0.853, 1.707]	0.266	1.342 [0.969, 1.859]	0.0731	1.300 [0.943, 1.792]	0.1019
	12−17	0.916 [0.592, 1.418]	0.6761	0.848 [0.550, 1.307]	0.4293	0.880 [0.574, 1.349]	0.5332
	18−29	1.547 [1.008, 2.374]	**0.0462**	1.360 [0.837, 2.212]	0.1969	1.461 [0.926, 2.304]	0.0968
	30−44	0.941 [0.684, 1.295]	0.6915	0.856 [0.622, 1.179]	0.318	0.900 [0.659, 1.228]	0.4805
	≥60	0.893 [0.707, 1.128]	0.3175	1.002 [0.779, 1.287]	0.9897	0.964 [0.765, 1.215]	0.7423
	45−59	Ref.		Ref.		Ref.	
Race/Hispanic origin	Non-Hispanic Black	0.805 [0.640, 1.013]	0.0621	0.929 [0.703, 1.227]	0.5795	0.909 [0.708, 1.167]	0.4281
	Hispanics	0.831 [0.710, 0.973]	**0.0242**	0.797 [0.675, 0.941]	**0.0109**	0.810 [0.693, 0.947]	**0.0114**
	Other/Multiracial	0.837 [0.658, 1.065]	0.1357	0.708 [0.521, 0.960]	**0.029**	0.773 [0.592, 1.009]	0.0575
	Non-Hispanic White	Ref.		Ref.		Ref.	
Education Attainment	Less than high school	1.604 [1.121, 2.293]	**0.0131**	1.718 [1.262, 2.339]	**0.002**	1.679 [1.213, 2.324]	**0.004**
	High school graduate	2.125 [1.544, 2.924]	**0.0001**	2.206 [1.578, 3.085]	**0.0001**	2.195 [1.603, 3.005]	**<0.0001**
	Some college (no degree)	1.175 [0.897, 1.537]	0.2223	1.329 [1.023, 1.727]	**0.0351**	1.27 [0.98, 1.646]	0.0678
	Bachelor’s degree or above	Ref.		Ref.		Ref.	

COT = cotinine; HCT = trans-3′-hydroxycotinine; TNE2 = (total cotinine/176.2151) + (total trans-3′-hydroxycotinine/192.2145); CI = confidence interval; −sCOT = non-users with undetectable serum COT; +sCOT = non-users with detectable serum COT. The statistical significance of regression coefficients is highlighted in bold.

**Table 5:** Sample-weighted log-linear regression results for urinary cotinine (µg/L) and TNEs (µmol/L) on frequency of tobacco usage and demographic factors among people who use tobacco, from NHANES 2015–2016.

Factor	Level	COT	*p*-Value	TNE2	*p*-Value	TNE3	*p*-Value	TNE7	*p*-Value
		Exponentiated Coefficient (95% CI)		Exponentiated Coefficient (95% CI)		Exponentiated Coefficient (95% CI)		Exponentiated Coefficient (95% CI)	
Intercept		1936 [1296, 2892]	**<0.0001**	24.88 [15.28, 40.49]	**<0.0001**	31.81 [21.58, 46.90]	**<0.0001**	36.18 [24.82, 52.74]	**<0.0001**
Creatinine, urine		1.007 [1.005, 1.009]	**<0.0001**	1.009 [1.006, 1.011]	**<0.0001**	1.008 [1.007, 1.010]	**<0.0001**	1.008 [1.007, 1.010]	**<0.0001**
User	Non-daily	0.516 [0.401, 0.664]	**<0.0001**	0.538 [0.420, 0.688]	**<0.0001**	0.505 [0.402, 0.635]	**<0.0001**	0.501 [0.400, 0.627]	**<0.0001**
	Daily	Ref.		Ref.		Ref.		Ref.	
Sex	Female	0.951 [0.829, 1.092]	0.4523	0.984 [0.804, 1.203]	0.8654	1.035 [0.888, 1.206]	0.6398	1.045 [0.902, 1.211]	0.5336
	Male	Ref.		Ref.		Ref.		Ref.	
Age	18−29	0.365 [0.260, 0.514]	**<0.0001**	0.327 [0.237, 0.451]	**<0.0001**	0.325 [0.238, 0.444]	**<0.0001**	0.326 [0.240, 0.445]	**<0.0001**
	30−44	0.703 [0.524, 0.944]	**0.0222**	0.640 [0.478, 0.856]	**0.0051**	0.650 [0.487, 0.868]	**0.0063**	0.646 [0.481, 0.867]	**0.0065**
	≥60	0.716 [0.528, 0.971]	**0.0337**	0.723 [0.531, 0.984]	**0.0406**	0.706 [0.522, 0.954]	**0.0264**	0.718 [0.533, 0.966]	**0.0309**
	45−59	Ref.		Ref.		Ref.		Ref.	
Race/Hispanic origin	Non-Hispanic Black	0.443 [0.370, 0.531]	**<0.0001**	0.516 [0.433, 0.616]	**<0.0001**	0.512 [0.437, 0.601]	**<0.0001**	0.525 [0.450, 0.613]	**<0.0001**
	Hispanics	0.543 [0.393, 0.751]	**0.0011**	0.531 [0.360, 0.784]	**0.0035**	0.527 [0.374, 0.742]	**0.0012**	0.530 [0.381, 0.736]	**0.0009**
	Other/Multiracial	0.747 [0.570, 0.979]	**0.036**	0.640 [0.454, 0.903]	**0.0144**	0.650 [0.472, 0.897]	**0.012**	0.659 [0.482, 0.903]	**0.0128**
	Non-Hispanic White	Ref.		Ref.		Ref.		Ref.	
Education Attainment	Less than high school	1.322 [0.958, 1.824]	0.0842	1.297 [0.892, 1.886]	0.1587	1.274 [0.947, 1.714]	0.1028	1.277 [0.955, 1.709]	0.0931
	High school graduate	1.064 [0.775, 1.461]	0.6828	1.085 [0.805, 1.463]	0.5671	1.039 [0.809, 1.334]	0.7506	1.030 [0.800, 1.327]	0.8042
	Some college (no degree)	1.014 [0.694, 1.480]	0.94	1.098 [0.744, 1.619]	0.6158	1.030 [0.755, 1.407]	0.8402	1.019 [0.753, 1.381]	0.8941
	Bachelor’s degree or above	Ref.		Ref.		Ref.		Ref.	

COT = cotinine; TNE2 = (total cotinine/176.2151) + (total trans-3′-hydroxycotinine/192.2145); TNE3 = (total nicotine/162.2316) + (total cotinine/176.2151) + (total trans-3′-hydroxycotinine/192.2145); TNE7 = (total nicotine/162.2316) + (total cotinine/176.2151) + (total trans-3′-hydroxycotinine/192.2145) + (total cotinine N-oxide/192.2145) + (total nicotine 1′-oxide/178.231) + (total 1-(3-pyridyl)-1-butanol-4-carboxylic acid/181.1885) + (total nornicotine/148.2050); CI = confidence interval. The statistical significance of regression coefficients is highlighted in bold.

## Data Availability

The datasets generated in this study are available in NHANES Questionnaires, Datasets and Related Documentation: UCOT_I, COT_I [44].

## Supplementary Materials

The following supporting information can be downloaded at: https://www.mdpi.com/article/10.3390/ijerph19063660/s1, Table S1: Sample-weighted geometric mean for cotinine, trans-3′-hydroxycotinine (µg/L) and TNE2 (µmol/L), with 95% confidence interval, among non-users, from NHANES 2015–2016; Table S2: Sample-weighted geometric mean for cotinine and trans-3′-hydroxycotinine (µg/L) and TNE2, TNE3, TNE7 (µmol/L), with 95% confidence interval, among people who use tobacco, from NHANES 2015–2016; Table S3: Pairwise comparisons of log-10 transformed urinary cotinine (µg/L), trans-3′-hydroxycotinine (µg/L) and TNE2 (µmol/L) least-square mean ratios between groups of non-users, from NHANES 2015–2016. Bonferroni adjustment was used to correct for multiple comparisons; Table S4: Pairwise comparisons of log-10 transformed urinary cotinine (µg/L) and TNEs (µmol/L) least-square mean ratios between groups of users, from NHANES 2015–2016. Bonferroni adjustment was used to correct for multiple comparisons; Table S5: Sample-weighted log-linear regression results for urinary cotinine (µg/L), trans-3′-hydroxycotinine (µg/L) and TNE2 (µmol/L) on extent of passive exposure to nicotine and demographic factors among non-users, excluding participants younger than 25 years; Table S6: Sample-weighted log-linear regression results for urinary cotinine (µg/L) and TNEs (µmol/L) on frequency of tobacco usage and demographic factors among people who use tobacco, excluding participants younger than 25 years.
