# Supplementary material for: Nicotine Exposure in the U.S. Population: Total Urinary Nicotine Biomarkers in NHANES 2015–2016
Source: Int J Environ Res Public Health. 2022 Mar 19;19(6):3660. doi: 10.3390/ijerph19063660 (PMC8955498; doi:10.3390/ijerph19063660)
Supplement: Supplementary file 1 [file ijerph-19-03660-s001.zip › ijerph-1603508-supplementary.pdf]

International Journal of *Environmental Research and Public Health*

MDPI

**Table S1.** Sample-weighted geometric mean for cotinine, *trans*-3′-hydroxycotinine (μg/L) and TNE2 (μmol/L), with 95% confidence interval, among non-users, from NHANES 2015–2016.

| | **−sCOT** | | | **+sCOT** | | | **All non-users** | | |
|---|---|---|---|---|---|---|---|---|---|
| | **COT** | **HCT** | **TNE2** | **COT** | **HCT** | **TNE2** | **COT** | **HCT** | **TNE2** |
| All | 0.0974<br>[0.0908, 0.105] | 0.164<br>[0.150, 0.181] | 0.00147<br>[0.00136, 0.00158] | 0.854<br>[0.700, 1.04] | 1.52<br>[1.25, 1.86] | 0.0133<br>[0.0109, 0.0162] | 0.303<br>[0.254, 0.361] | 0.526<br>[0.443, 0.624] | 0.00464<br>[0.00389, 0.00552] |
| 6-11 | 0.0830<br>[0.0680, 0.102] | 0.162<br>[0.129, 0.203] | 0.00137<br>[0.00111, 0.00168] | 1.14<br>[0.850, 1.52] | 2.08<br>[1.63, 2.65] | 0.0180<br>[0.0139, 0.0233] | 0.414<br>[0.331, 0.519] | 0.778<br>[0.611, 0.990] | 0.00666<br>[0.00529, 0.00837] |
| 12-17 | 0.0900<br>[0.0700, 0.116] | 0.146<br>[0.0990, 0.214] | 0.00133<br>[0.00097, 0.00183] | 1.07<br>[0.530, 2.17] | 1.84<br>[0.880, 3.85] | 0.0162<br>[0.00783, 0.0335] | 0.455<br>[0.276, 0.751] | 0.765<br>[0.458, 1.28] | 0.00682<br>[0.00411, 0.0113] |
| 18-29 | 0.136<br>[0.113, 0.164] | 0.200<br>[0.166, 0.240] | 0.00190<br>[0.00160, 0.00226] | 1.38<br>[0.727, 2.63] | 2.27<br>[1.12, 4.60] | 0.0207<br>[0.0105, 0.0407] | 0.606<br>[0.382, 0.963] | 0.956<br>[0.574, 1.59] | 0.00885<br>[0.00542, 0.0145] |
| 30-44 | 0.100<br>[0.0870, 0.115] | 0.149<br>[0.117, 0.188] | 0.00140<br>[0.00117, 0.00168] | 0.643<br>[0.457, 0.905] | 1.10<br>[0.770, 1.56] | 0.00975<br>[0.00684, 0.0139] | 0.259<br>[0.218, 0.308] | 0.413<br>[0.334, 0.511] | 0.00379<br>[0.00311, 0.00461] |
| 45-59 | 0.0970<br>[0.0820, 0.113] | 0.162<br>[0.127, 0.205] | 0.00143<br>[0.00117, 0.00175] | 0.649<br>[0.361, 1.17] | 1.17<br>[0.660, 2.07] | 0.0101<br>[0.00569, 0.0179] | 0.219<br>[0.141, 0.340] | 0.378<br>[0.242, 0.592] | 0.00331<br>[0.00212, 0.00515] |
| ≥60 | 0.0900<br>[0.0810, 0.0990] | 0.172<br>[0.151, 0.195] | 0.00146<br>[0.00131, 0.00162] | 0.676<br>[0.449, 1.02] | 1.35<br>[0.912, 1.99] | 0.0112<br>[0.00753, 0.0166] | 0.222<br>[0.168, 0.293] | 0.432<br>[0.333, 0.562] | 0.00363<br>[0.00278, 0.00475] |
| Non-Hispanic White | 0.0930<br>[0.0840, 0.103] | 0.159<br>[0.138, 0.183] | 0.00140<br>[0.00125, 0.00157] | 0.876<br>[0.651, 1.18] | 1.58<br>[1.15, 2.16] | 0.0136<br>[0.0100, 0.0184] | 0.283<br>[0.222, 0.360] | 0.496<br>[0.391, 0.630] | 0.00433<br>[0.00341, 0.00550] |
| Non-Hispanic Black | 0.110<br>[0.0930, 0.129] | 0.241<br>[0.203, 0.286] | 0.00198<br>[0.00171, 0.00229] | 0.970<br>[0.767, 1.23] | 2.07<br>[1.59, 2.70] | 0.0174<br>[0.0136, 0.0222] | 0.487<br>[0.367, 0.645] | 1.05<br>[0.768, 1.43] | 0.00876<br>[0.00651, 0.0118] |
| Hispanic | 0.107<br>[0.0940, 0.121] | 0.173<br>[0.148, 0.202] | 0.00156<br>[0.00135, 0.00179] | 0.930<br>[0.762, 1.13] | 1.59<br>[1.30, 1.95] | 0.0141<br>[0.0116, 0.0172] | 0.299<br>[0.246, 0.363] | 0.498<br>[0.395, 0.628] | 0.00445<br>[0.00358, 0.00552] |
| Other/Multiracial | 0.103<br>[0.0930, 0.113] | 0.144<br>[0.119, 0.174] | 0.00140<br>[0.00121, 0.00163] | 0.595<br>[0.413, 0.855] | 0.885<br>[0.566, 1.38] | 0.00842<br>[0.00563, 0.0126] | 0.302<br>[0.226, 0.404] | 0.440<br>[0.310, 0.625] | 0.00422<br>[0.00307, 0.00581] |
| Male | 0.105<br>[0.0920, 0.120] | 0.182<br>[0.158, 0.211] | 0.00160<br>[0.00139, 0.00183] | 1.02<br>[0.750, 1.38] | 1.77<br>[1.31, 2.39] | 0.0155<br>[0.0115, 0.0210] | 0.387<br>[0.298, 0.501] | 0.671<br>[0.517, 0.871] | 0.00589<br>[0.00453, 0.00765] |
| Female | 0.0920<br>[0.0860, 0.0990] | 0.153<br>[0.136, 0.173] | 0.00138<br>[0.00126, 0.00151] | 0.715<br>[0.589, 0.866] | 1.30<br>[1.06, 1.61] | 0.0113<br>[0.00923, 0.0138] | 0.246<br>[0.210, 0.288] | 0.427<br>[0.364, 0.501] | 0.00378<br>[0.00323, 0.00443] |
| Less than high school | 0.0970<br>[0.0850, 0.109] | 0.173<br>[0.143, 0.209] | 0.00150<br>[0.00129, 0.00175] | 1.07<br>[0.758, 1.50] | 1.86<br>[1.33, 2.59] | 0.0164<br>[0.0117, 0.0229] | 0.408<br>[0.309, 0.539] | 0.720<br>[0.542, 0.956] | 0.00631<br>[0.00477, 0.00834] |
| High school graduate | 0.107<br>[0.0890, 0.130] | 0.170<br>[0.137, 0.212] | 0.00158<br>[0.00130, 0.00191] | 1.84<br>[1.20, 2.84] | 3.47<br>[2.23, 5.41] | 0.0297<br>[0.0192, 0.0459] | 0.653<br>[0.454, 0.939] | 1.15<br>[0.768, 1.74] | 0.0102<br>[0.00690, 0.0150] |
| Some college (no degree) | 0.105<br>[0.0830, 0.134] | 0.190<br>[0.154, 0.234] | 0.00165<br>[0.00133, 0.00204] | 0.667<br>[0.479, 0.927] | 1.28<br>[0.915, 1.79] | 0.0109<br>[0.00783, 0.0151] | 0.285<br>[0.207, 0.393] | 0.532<br>[0.392, 0.723] | 0.00456<br>[0.00334, 0.00623] |
| Bachelor's degree or above | 0.0910<br>[0.0790, 0.103] | 0.144<br>[0.119, 0.175] | 0.00131<br>[0.00111, 0.00155] | 0.440<br>[0.290, 0.669] | 0.702<br>[0.465, 1.06] | 0.00636<br>[0.00423, 0.00958] | 0.164<br>[0.134, 0.201] | 0.262<br>[0.208, 0.329] | 0.00238<br>[0.00192, 0.00294] |

COT = cotinine; HCT = *trans*-3′-hydroxycotinine; TNE2 = (total cotinine/176.2151) + (total *trans*-3′-hydroxycotinine/192.2145); −sCOT = non-users with undetectable serum COT; +sCOT = non-users with detectable serum COT.

**Table S2.** Sample-weighted geometric mean for cotinine and *trans*-3′-hydroxycotinine (µg/L) and TNE2, TNE3, TNE7 (µmol/L), with 95% confidence interval, among people who use tobacco, from NHANES 2015–2016.

| | **COT** | **HCT** | **TNE2** | **TNE3** | **TNE7** |
|---|---|---|---|---|---|
| All | 2169 [1972, 2385] | 3880 [3412, 4412] | 34.3 [30.9, 38.2] | 41.6 [37.5, 46.2] | 47.4 [42.8, 52.4] |
| 18-29 | 1049 [805, 1366] | 1725 [1298, 2292] | 16.3 [12.3, 21.6] | 19.3 [14.3, 26.0] | 22.0 [16.3, 29.8] |
| 30-44 | 2205 [1706, 2851] | 3821 [2844, 5135] | 33.9 [25.8, 44.7] | 41.6 [31.8, 54.4] | 47.0 [36.1, 61.1] |
| 45-59 | 3167 [2785, 3603] | 6035 [4933, 7383] | 51.6 [44.4, 60.1] | 63.0 [54.6, 72.6] | 71.6 [62.1, 82.6] |
| ≥60 | 2655 [2324, 3033] | 5040 [4057, 6261] | 43.3 [37.1, 50.7] | 52.0 [44.2, 61.1] | 60.2 [51.5, 70.5] |
| Non-Hispanic White | 2769 [2443, 3137] | 4916 [4101, 5894] | 43.1 [37.0, 50.3] | 52.4 [45.3, 60.8] | 59.4 [51.3, 68.8] |
| Non-Hispanic Black | 1364 [1175, 1583] | 3061 [2268, 4131] | 26.0 [21.0, 32.2] | 31.1 [25.2, 38.4] | 36.1 [29.4, 44.5] |
| Hispanic | 1103 [814, 1494] | 1779 [1160, 2728] | 16.4 [11.8, 22.9] | 19.5 [14.1, 27.0] | 22.2 [16.1, 30.6] |
| Other/Multiracial | 1544 [1129, 2112] | 1916 [1339, 2741] | 19.5 [14.3, 26.7] | 24.3 [17.8, 33.2] | 27.9 [20.5, 38.0] |
| Male | 2388 [2066, 2761] | 4372 [3741, 5111] | 38.1 [33.0, 44.1] | 45.1 [39.0, 52.2] | 51.2 [44.4, 59.1] |
| Female | 1847 [1638, 2082] | 3180 [2443, 4138] | 28.9 [23.7, 35.1] | 36.3 [30.5, 43.1] | 41.5 [35.0, 49.3] |
| Less than high school | 2597 [2077, 3246] | 4183 [3359, 5208] | 39.0 [32.1, 47.4] | 48.5 [40.0, 58.6] | 55.8 [46.1, 67.5] |
| High school graduate | 2427 [1948, 3025] | 4320 [3235, 5770] | 38.5 [30.1, 49.3] | 46.3 [36.7, 58.6] | 52.6 [41.8, 66.2] |
| Some college (no degree) | 1854 [1418, 2424] | 3686 [2867, 4740] | 30.8 [23.8, 39.9] | 36.4 [28.2, 47.0] | 41.2 [32.1, 52.9] |
| Bachelor's degree or above | 1901 [1383, 2612] | 3205 [2067, 4969] | 29.2 [20.0, 42.5] | 36.4 [26.9, 49.3] | 41.6 [30.9, 56.1] |
| Daily | 2842 [2496, 3236] | 4954 [4238, 5790] | 43.9 [38.5, 50.1] | 54.5 [47.9, 61.9] | 62.3 [54.9, 70.7] |
| Non-daily | 1383 [1005, 1903] | 2585 [1773, 3769] | 22.8 [16.2, 32.2] | 26.5 [19.0, 37.0] | 30.0 [21.6, 41.7] |

COT = cotinine; HCT = *trans*-3′-hydroxycotinine; TNE2 = (total cotinine/176.2151) + (total *trans*-3′-hydroxycotinine/192.2145); TNE3 = (total nicotine/162.2316) + (total cotinine/176.2151) + (total *trans*-3′-hydroxycotinine/192.2145); TNE7 = (total nicotine/162.2316) + (total cotinine/176.2151) + (total *trans*-3′-hydroxycotinine/192.2145) + (total cotinine N-oxide/192.2145) + (total nicotine 1′-oxide/178.231) + (total 1-(3-pyridyl)-1-butanol-4-carboxylic acid/181.1885) + (total nornicotine/148.2050).

**Table S3.** Pairwise comparisons of log-10 transformed urinary cotinine (µg/L), *trans*-3′-hydroxycotinine (µg/L) and TNE2 (µmol/L) least-square mean ratios between groups of non-users, from NHANES 2015–2016. Bonferroni adjustment was used to correct for multiple comparisons.

| | | | COT | | HCT | | TNE2 | |
|---|---|---|---|---|---|---|---|---|
| | **Group A** | **Group B** | **Ratio A/B [95% CI]** | **Adjusted *p*-Value** | **Ratio A/B [95% CI]** | **Adjusted *p*-Value** | **Ratio A/B [95% CI]** | **Adjusted *p*-Value** |
| Non-user | -sCOT | +sCOT | 0.143 [0.118, 0.173] | **<0.0001** | 0.137 [0.112, 0.168] | **<0.0001** | 0.140 [0.116, 0.169] | **<0.0001** |
| Sex | Female | Male | 0.946 [0.799, 1.122] | 0.4998 | 0.987 [0.824, 1.182] | 0.8760 | 0.979 [0.821, 1.166] | 0.7957 |
| Age | 12-17 | 6-11 | 0.759 [0.453, 1.272] | >0.9999 | 0.632 [0.375, 1.064] | 0.1174 | 0.677 [0.404, 1.133] | 0.2793 |
| | | 18-29 | 0.592 [0.240, 1.460] | 0.9212 | 0.623 [0.241, 1.612] | >0.9999 | 0.602 [0.241, 1.506] | >0.9999 |
| | | 30-44 | 0.974 [0.340, 2.789] | >0.9999 | 0.990 [0.353, 2.780] | >0.9999 | 0.978 [0.346, 2.763] | >0.9999 |
| | | 45-59 | 0.916 [0.449, 1.870] | >0.9999 | 0.848 [0.418, 1.719] | >0.9999 | 0.880 [0.437, 1.770] | >0.9999 |
| | | ≥60 | 1.026 [0.451, 2.335] | >0.9999 | 0.847 [0.402, 1.785] | >0.9999 | 0.912 [0.421, 1.976] | >0.9999 |
| | 18-29 | 6-11 | 1.282 [0.610, 2.692] | >0.9999 | 1.013 [0.528, 1.945] | >0.9999 | 1.124 [0.568, 2.223] | >0.9999 |
| | | 30-44 | 1.644 [0.811, 3.330] | 0.4037 | 1.589 [0.701, 3.603] | >0.9999 | 1.623 [0.755, 3.493] | 0.6541 |
| | | 45-59 | 1.547 [0.769, 3.115] | 0.6933 | 1.360 [0.615, 3.010] | >0.9999 | 1.461 [0.693, 3.077] | >0.9999 |
| | | ≥60 | 1.733 [0.861, 3.488] | 0.2289 | 1.358 [0.641, 2.878] | >0.9999 | 1.515 [0.734, 3.124] | 0.9621 |
| | 30-44 | 6-11 | 0.780 [0.368, 1.654] | >0.9999 | 0.638 [0.309, 1.316] | 0.7082 | 0.692 [0.335, 1.429] | >0.9999 |
| | | 45-59 | 0.941 [0.559, 1.586] | >0.9999 | 0.856 [0.507, 1.445] | >0.9999 | 0.900 [0.541, 1.497] | >0.9999 |
| | | ≥60 | 1.054 [0.722, 1.539] | >0.9999 | 0.855 [0.599, 1.221] | >0.9999 | 0.933 [0.654, 1.332] | >0.9999 |
| | 6-11 | 45-59 | 1.207 [0.685, 2.128] | >0.9999 | 1.342 [0.788, 2.286] | >0.9999 | 1.300 [0.769, 2.196] | >0.9999 |
| | | ≥60 | 1.352 [0.741, 2.465] | >0.9999 | 1.340 [0.806, 2.230] | 0.9498 | 1.348 [0.791, 2.296] | >0.9999 |
| | ≥60 | 45-59 | 0.893 [0.610, 1.308] | >0.9999 | 1.002 [0.665, 1.509] | >0.9999 | 0.964 [0.661, 1.408] | >0.9999 |
| Race/Hispanic origin | Hispanic | Non-Hispanic Black | 1.032 [0.770, 1.383] | >0.9999 | 0.858 [0.585, 1.260] | >0.9999 | 0.891 [0.635, 1.250] | >0.9999 |
| | | Other/Multiracial | 0.992 [0.734, 1.341] | >0.9999 | 1.127 [0.786, 1.614] | >0.9999 | 1.048 [0.758, 1.449] | >0.9999 |
| | | Non-Hispanic White | 0.831 [0.664, 1.040] | 0.1454 | 0.797 [0.629, 1.010] | 0.0653 | 0.810 [0.649, 1.012] | 0.0687 |
| | Non-Hispanic Black | Other/Multiracial | 0.961 [0.647, 1.430] | >0.9999 | 1.313 [0.787, 2.188] | 0.7625 | 1.176 [0.750, 1.843] | >0.9999 |
| | | Non-Hispanic White | 0.805 [0.581, 1.116] | 0.3727 | 0.929 [0.625, 1.381] | >0.9999 | 0.909 [0.636, 1.298] | >0.9999 |
| | Other/Multiracial | Non-Hispanic White | 0.837 [0.595, 1.179] | 0.8143 | 0.708 [0.458, 1.093] | 0.1740 | 0.773 [0.529, 1.130] | 0.3449 |
| Education Attainment | Less than high school | High school graduate | 0.755 [0.486, 1.172] | 0.4283 | 0.779 [0.512, 1.184] | 0.5402 | 0.765 [0.505, 1.159] | 0.4155 |
| | | Some college (no degree) | 1.365 [0.798, 2.336] | 0.5932 | 1.292 [0.812, 2.058] | 0.6880 | 1.322 [0.812, 2.154] | 0.6162 |
| | | Bachelor's degree or above | 1.604 [0.963, 2.670] | 0.0787 | 1.718 [1.107, 2.666] | **0.0118** | 1.679 [1.057, 2.668] | **0.0237** |
| | High school graduate | Some college (no degree) | 1.809 [1.183, 2.767] | **0.0043** | 1.660 [1.080, 2.550] | **0.0163** | 1.728 [1.150, 2.598] | **0.0060** |
| | | Bachelor's degree or above | 2.125 [1.348, 3.349] | **0.0009** | 2.206 [1.369, 3.557] | **0.0009** | 2.195 [1.403, 3.434] | **0.0005** |
| | Some college (no degree) | Bachelor's degree or above | 1.175 [0.800, 1.724] | >0.9999 | 1.329 [0.915, 1.930] | 0.2104 | 1.270 [0.878, 1.837] | 0.4066 |

COT = cotinine; HCT = *trans*-3′-hydroxycotinine; TNE2 = (total cotinine/176.2151) + (total *trans*-3′-hydroxycotinine/192.2145); CI = confidence interval; -sCOT = non-users with undetectable serum COT; +sCOT = non-users with detectable serum COT. The statistical significance of regression coefficients is highlighted in bold.

**Table S4.** Pairwise comparisons of log-10 transformed urinary cotinine (μg/L) and TNEs (μmol/L) least-square mean ratios between groups of users, from NHANES 2015–2016. Bonferroni adjustment was used to correct for multiple comparisons.

| | | | COT | | TNE2 | | TNE3 | | TNE7 | |
|---|---|---|---|---|---|---|---|---|---|---|
| | **Group A** | **Group B** | **Ratio A/B [95% CI]** | **Adjusted *p*-Value** | **Ratio A/B [95% CI]** | **Adjusted *p*-Value** | **Ratio A/B [95% CI]** | **Adjusted *p*-Value** | **Ratio A/B [95% CI]** | **Adjusted *p*-Value** |
| User | Non-daily | Daily | 0.516 [0.401, 0.664] | **<0.0001** | 0.538 [0.402, 0.688] | **<0.0001** | 0.505 [0.402, 0.635] | **<0.0001** | 0.501 [0.400, 0.627] | **<0.0001** |
| Sex | Female | Male | 0.951 [0.829, 1.092] | 0.4523 | 0.984 [0.804, 1.203] | 0.8654 | 1.035 [0.888, 1.206] | 0.6398 | 1.045 [0.902, 1.211] | 0.5336 |
| Age | 18-29 | 30-44 | 0.519 [0.344, 0.783] | **0.0013** | 0.511 [0.330, 0.793] | **0.0019** | 0.500 [0.335, 0.746] | **0.0006** | 0.505 [0.341, 0.749] | **0.0006** |
| | | 45-59 | 0.365 [0.225, 0.594] | **<0.0001** | 0.327 [0.207, 0.517] | **<0.0001** | 0.325 [0.208, 0.507] | **<0.0001** | 0.326 [0.210, 0.507] | **<0.0001** |
| | | ≥60 | 0.510 [0.372, 0.699] | **<0.0001** | 0.453 [0.337, 0.608] | **<0.0001** | 0.460 [0.354, 0.599] | **<0.0001** | 0.455 [0.353, 0.586] | **<0.0001** |
| | 30-44 | 45-59 | 0.703 [0.462, 1.069] | 0.1331 | 0.640 [0.423, 0.968] | **0.0309** | 0.650 [0.431, 0.981] | **0.0376** | 0.646 [0.424, 0.983] | **0.0389** |
| | | ≥60 | 0.982 [0.565, 1.705] | >0.9999 | 0.885 [0.514, 1.525] | >0.9999 | 0.921 [0.541, 1.568] | >0.9999 | 0.900 [0.532, 1.522] | >0.9999 |
| | ≥60 | 45-59 | 0.716 [0.464, 1.105] | 0.2023 | 0.723 [0.466, 1.122] | 0.2435 | 0.706 [0.459, 1.085] | 0.1586 | 0.718 [0.470, 1.095] | 0.1854 |
| Race/ Hispanic origin | Hispanic | Non-Hispanic Black | 1.224 [0.699, 2.143] | >0.9999 | 1.029 [0.590, 1.795] | >0.9999 | 1.028 [0.639, 1.653] | >0.9999 | 1.008 [0.647, 1.573] | >0.9999 |
| | | Other/Multiracial | 0.727 [0.407, 1.296] | 0.6881 | 0.830 [0.414, 1.662] | >0.9999 | 0.810 [0.440, 1.490] | >0.9999 | 0.803 [0.444, 1.454] | >0.9999 |
| | | Non-Hispanic White | 0.543 [0.342, 0.862] | **0.0068** | 0.531 [0.305, 0.926] | **0.0210** | 0.527 [0.323, 0.858] | **0.0071** | 0.530 [0.331, 0.847] | **0.0055** |
| | Non-Hispanic Black | Other/Multiracial | 0.594 [0.335, 1.052] | 0.0864 | 0.806 [0.447, 1.454] | >0.9999 | 0.788 [0.457, 1.358] | >0.9999 | 0.797 [0.467, 1.359] | >0.9999 |
| | | Non-Hispanic White | 0.443 [0.343, 0.574] | **<0.0001** | 0.516 [0.401, 0.664] | **<0.0001** | 0.512 [0.408, 0.644] | **<0.0001** | 0.525 [0.421, 0.655] | **<0.0001** |
| | Other/Multiracial | Non-Hispanic White | 0.747 [0.509, 1.097] | 0.2162 | 0.640 [0.393, 1.044] | 0.0864 | 0.650 [0.412, 1.027] | 0.0720 | 0.659 [0.421, 1.032] | 0.0767 |
| Education Attainment | Less than high school | High school graduate | 1.243 [0.870, 1.776] | 0.5070 | 1.195 [0.834, 1.713] | 0.9153 | 1.226 [0.863, 1.742] | 0.5889 | 1.240 [0.870, 1.765] | 0.5096 |
| | | Some college (no degree) | 1.304 [0.965, 1.763] | 0.1037 | 1.182 [0.894, 1.562] | 0.5357 | 1.236 [0.973, 1.570] | 0.1006 | 1.253 [0.993, 1.581] | 0.0600 |
| | | Bachelor's degree or above | 1.322 [0.836, 2.091] | 0.5051 | 1.297 [0.761, 2.211] | 0.9522 | 1.274 [0.835, 1.943] | 0.6168 | 1.277 [0.844, 1.933] | 0.5586 |
| | High school graduate | Some college (no degree) | 1.050 [0.722, 1.526] | >0.9999 | 0.989 [0.682, 1.433] | >0.9999 | 1.008 [0.719, 1.412] | >0.9999 | 1.011 [0.722, 1.414] | >0.9999 |
| | | Bachelor's degree or above | 1.064 [0.677, 1.672] | >0.9999 | 1.085 [0.710, 1.660] | >0.9999 | 1.039 [0.727, 1.483] | >0.9999 | 1.030 [0.718, 1.478] | >0.9999 |
| | Some college (no degree) | Bachelor's degree or above | 1.014 [0.591, 1.737] | >0.9999 | 1.098 [0.631, 1.910] | >0.9999 | 1.030 [0.661, 1.606] | >0.9999 | 1.019 [0.662, 1.571] | >0.9999 |

COT = cotinine; HCT = *trans*-3′-hydroxycotinine; TNE2 = (total cotinine/176.2151) + (total *trans*-3′-hydroxycotinine/192.2145); TNE3 = (total nicotine/162.2316) + (total cotinine/176.2151) + (total *trans*-3′-hydroxycotinine/192.2145); TNE7 = (total nicotine/162.2316) + (total cotinine/176.2151) + (total *trans*-3′-hydroxycotinine/192.2145) + (total cotinine N-oxide/192.2145) + (total nicotine 1′-oxide/178.231) + (total 1-(3-pyridyl)-1-butanol-4-carboxylic acid/181.1885) + (total nornicotine/148.2050); CI = confidence interval. The statistical significance of regression coefficients is highlighted in bold.

**Table S5.** Sample-weighted log-linear regression results for urinary cotinine (μg/L), *trans*-3′-hydroxycotinine (μg/L) and TNE2 (μmol/L) on extent of passive exposure to nicotine and demographic factors among non-users, excluding participants younger than 25 years.

| Factor | Level | COT | | HCT | | TNE2 | |
|---|---|---|---|---|---|---|---|
| | | **Exponentiated Coefficient [95% CI]** | ***p*-Value** | **Exponentiated Coefficient [95% CI]** | ***p*-Value** | **Exponentiated Coefficient [95% CI]** | ***p*-Value** |
| Intercept | | 0.264 [0.163, 0.427] | **<0.0001** | 0.372 [0.233, 0.593] | **0.0004** | 0.003 [0.002, 0.005] | **<0.0001** |
| Creatinine, urine | | 1.007 [1.006, 1.009] | **<0.0001** | 1.009 [1.007, 1.010] | **<0.0001** | 1.008 [1.007, 1.010] | **<0.0001** |
| Non-user | -sCOT | 0.165 [0.129, 0.212] | **<0.0001** | 0.154 [0.125, 0.190] | **<0.0001** | 0.160 [0.128, 0.199] | **<0.0001** |
| | +sCOT | Ref. | | Ref. | | Ref. | |
| Sex | Female | 0.971 [0.786, 1.199] | 0.7678 | 1.045 [0.846, 1.290] | 0.6663 | 1.023 [0.829, 1.263] | 0.8205 |
| | Male | Ref. | | Ref. | | Ref. | |
| Age | 25-29 | 1.738 [1.126, 2.682] | **0.0160** | 1.458 [0.970, 2.193] | 0.0673 | 1.591 [1.062, 2.384] | **0.0272** |
| | 30-44 | 0.954 [0.689, 1.320] | 0.7594 | 0.868 [0.630, 1.196] | 0.3618 | 0.912 [0.667, 1.247] | 0.5384 |
| | ≥60 | 0.901 [0.717, 1.133] | 0.3485 | 1.015 [0.788, 1.308] | 0.8989 | 0.976 [0.774, 1.231] | 0.8269 |
| | 45-59 | Ref. | | Ref. | | Ref. | |
| Race/Hispanic origin | Non-Hispanic Black | 0.647 [0.498, 0.841] | **0.0030** | 0.744 [0.567, 0.977] | **0.0351** | 0.735 [0.565, 0.957] | **0.0251** |
| | Hispanics | 0.864 [0.709, 1.051] | 0.1330 | 0.819 [0.628, 1.069] | 0.1305 | 0.837 [0.662, 1.059] | 0.1278 |
| | Other/Multiracial | 0.794 [0.619, 1.018] | 0.0661 | 0.637 [0.475, 0.856] | **0.0053** | 0.714 [0.549, 0.929] | **0.0155** |
| | Non-Hispanic White | Ref. | | Ref. | | Ref. | |
| Education Attainment | Less than high school | 1.659 [1.055, 2.607] | **0.0306** | 1.749 [1.153, 2.654] | **0.0120** | 1.724 [1.121, 2.653] | **0.0166** |
| | High school graduate | 1.937 [1.439, 2.607] | **0.0003** | 1.905 [1.411, 2.570] | **0.0004** | 1.941 [1.460, 2.581] | **0.0002** |
| | Some college (no degree) | 1.192 [0.919, 1.546] | 0.1710 | 1.322 [1.021, 1.710] | **0.0357** | 1.272 [0.988, 1.637] | 0.0604 |
| | Bachelor's degree or above | Ref. | | Ref. | | Ref. | |

COT = cotinine; HCT = *trans*-3′-hydroxycotinine; TNE2 = (total cotinine/176.2151) + (total *trans*-3′-hydroxycotinine/192.2145); CI = confidence interval; -sCOT = non-users with undetectable serum COT; +sCOT = non-users with detectable serum COT. The statistical significance of regression coefficients is highlighted in bold.

**Table S6.** Sample-weighted log-linear regression results for urinary cotinine (µg/L) and TNEs (µmol/L) on frequency of tobacco usage and demographic factors among people who use tobacco, excluding participants younger than 25 years.

| Factor | Level | COT | | TNE2 | | TNE3 | | TNE7 | |
|---|---|---|---|---|---|---|---|---|---|
| | | **Exponentiated Coefficient [95% CI]** | ***p*-Value** | **Exponentiated Coefficient [95% CI]** | ***p*-Value** | **Exponentiated Coefficient [95% CI]** | ***p*-Value** | **Exponentiated Coefficient [95% CI]** | ***p*-Value** |
| Intercept | | 1809 [1219, 2684] | **<0.0001** | 23.09 [14.08, 37.86] | **<0.0001** | 30.83 [20.79, 45.72] | **<0.0001** | 35.35 [24.14, 51.76] | **<0.0001** |
| Creatinine, urine | | 1.007 [1.005, 1.010] | **<0.0001** | 1.009 [1.006, 1.011] | **<0.0001** | 1.008 [1.006, 1.010] | **<0.0001** | 1.008 [1.007, 1.010] | **<0.0001** |
| User | Non-daily | 0.550 [0.424, 0.713] | **0.0002** | 0.583 [0.453, 0.749] | **0.0004** | 0.536 [0.431, 0.665] | **<0.0001** | 0.529 [0.429, 0.653] | **<0.0001** |
| | Daily | Ref. | | Ref. | | Ref. | | Ref. | . |
| Sex | Female | 0.935 [0.810, 1.078] | 0.3300 | 0.972 [0.807, 1.171] | 0.7510 | 0.998 [0.861, 1.156] | 0.9729 | 1.002 [0.870, 1.155] | 0.9767 |
| | Male | Ref. | | Ref. | | Ref. | | Ref. | |
| Age | 25-29 | 0.419 [0.306, 0.572] | **<0.0001** | 0.392 [0.285, 0.540] | **<0.0001** | 0.383 [0.284, 0.517] | **<0.0001** | 0.381 [0.283, 0.514] | **<0.0001** |
| | 30-44 | 0.694 [0.517, 0.932] | **0.0184** | 0.631 [0.473, 0.842] | **0.0040** | 0.644 [0.482, 0.859] | **0.0053** | 0.640 [0.477, 0.859] | **0.0056** |
| | ≥60 | 0.725 [0.533, 0.985] | **0.0409** | 0.733 [0.543, 0.988] | **0.0426** | 0.710 [0.530, 0.951] | **0.0247** | 0.722 [0.541, 0.962] | **0.0289** |
| | 45-59 | Ref. | | Ref. | | Ref. | | Ref. | |
| Race/Hispanic origin | Non-Hispanic Black | 0.439 [0.369, 0.523] | **<0.0001** | 0.517 [0.431, 0.621] | **<0.0001** | 0.509 [0.429, 0.603] | **<0.0001** | 0.522 [0.443, 0.616] | **<0.0001** |
| | Hispanics | 0.546 [0.420, 0.711] | **0.0002** | 0.532 [0.393, 0.721] | **0.0005** | 0.518 [0.384, 0.699] | **0.0003** | 0.522 [0.390, 0.698] | **0.0002** |
| | Other/Multiracial | 0.832 [0.641, 1.078] | 0.1510 | 0.686 [0.477, 0.986] | **0.0429** | 0.698 [0.500, 0.973] | **0.0358** | 0.704 [0.505, 0.980] | **0.0390** |
| | Non-Hispanic White | Ref. | | Ref. | | Ref. | | Ref. | |
| Education Attainment | Less than high school | 1.312 [0.920, 1.871] | 0.1234 | 1.342 [0.865, 2.082] | 0.1738 | 1.294 [0.923, 1.813] | 0.1245 | 1.292 [0.928, 1.798] | 0.1202 |
| | High school graduate | 1.116 [0.843, 1.477] | 0.4170 | 1.151 [0.874, 1.516] | 0.2925 | 1.082 [0.871, 1.345] | 0.4515 | 1.071 [0.861, 1.331] | 0.5136 |
| | Some college (no degree) | 1.055 [0.750, 1.484] | 0.7442 | 1.151 [0.796, 1.666] | 0.4293 | 1.079 [0.801, 1.452] | 0.5954 | 1.064 [0.795, 1.424] | 0.6577 |
| | Bachelor's degree or above | Ref. | | Ref. | | Ref. | | Ref. | |

COT = cotinine; HCT = *trans*-3′-hydroxycotinine; TNE2 = (total cotinine/176.2151) + (total *trans*-3′-hydroxycotinine/192.2145); TNE3 = (total nicotine/162.2316) + (total cotinine/176.2151) + (total *trans*-3′-hydroxycotinine/192.2145); TNE7 = (total nicotine/162.2316) + (total cotinine/176.2151) + (total *trans*-3′-hydroxycotinine/192.2145) + (total cotinine N-oxide/192.2145) + (total nicotine 1′-oxide/178.231) + (total 1-(3-pyridyl)-1-butanol-4-carboxylic acid/181.1885) + (total nornicotine/148.2050); CI = confidence interval. The statistical significance of regression coefficients is highlighted in bold.
